# 

**Published:** 1878-10

**Authors:** 


### ARTICLE V.

### *Virginia State Dental Association.*

The regular annual meeting of the Virginia State Dental Association will be held in the City of Richmond on the second Monday in December, 1878.

It is deemed highly important for the interest of the profession throughout the State, that this meeting should be as full as possible.

A cordial invitation is hereby extended, not only to the members of the society, but to all members of the profession, whether residing in this or other States, to meet with us.

Those residing in this State, and not members of the Society, are urgently invited to come and unite with us, thus lending their aid in carrying forward the enterprises which have for their end the good and welfare of the whole profession.

The members of the regular standing committees are earnestly urged to prepare as full and complete reports as possible, experience having shown that the more faithfully the committees perform the duties assigned them, the more interesting and useful our meetings are made.

The duties of the Standing Committees are set forth in the following articles of the Constitution:

ARTICLE I.

SEC. 1. It shall be the duty of each member of a committee to make an individual report, so far as such report *can* be made, and in case of inability to be present at the meeting at which such report is due, to forward it to the Recor ding Secretary, from whom it may be obtained by the Chairman of each Committee respectively at the time of the assembling of the Association.

Sec. 2. Each committee shall report, if practicable, the results of *original* investigations in their several departments, and also such *new* matter collected from all sources at their command as may be of interest and profit to the Association.

Sec. 3. The Committee on Operative and Mechanical Dentistry shall thoroughly test and report upon all *new* models and materials, and upon their physical properties, stating clearly *why* any particular material or mode of practice should claim attention, and giving tabulated lists of successes and failures, so far as may be attainable.

The resident Executive Committee is charged with the duty of providing entertainments for non-resident members, and visitors during their stay in the city, and from the well known energy and enterprise of the gentlemen composing that committee, we have good reason to believe that their portion of the programme will be carried out to the satisfaction of all.

Regular Standing Committees for the Year 1878.

*Committee on Publication.*—Dr. L. M. Cowardin, Richmond; Dr. G. Smith, Louisa C. H.; Dr. D. B. Garden, Farmville.

*Committee on Operative Dentistry.*—Dr. S. H. Henkel, Staunton; Dr. John W. Scribner, Charlottesville; Dr. Wm. Farmer, Wytheville.

*Committee on Mechanical Dentistry.*—Dr. G. A. Sprinkle, Culpeper C. H.; Prof. J. B. Hodgkin, Washington D. C.; Dr. W. E. Norris, Charlottesville.

*Committee on Dental Education and Literature.*—Dr. George H. Chewning, Fredericksburg; Dr. Frank L. Harris, Staunton; Dr. S. G. Cowardin, Richmond.

*Committee on Dental Pathology.*—Dr. N. M. Burkholder, Harrisonburg; Dr. Jud. B. Wood, Richmond; Dr. Jas. F. Thompson, Fredericksburg.

*Committee on Physiology, Histology, Microscopy and Chemistry.*—Dr. W. W. H. Thackston, Farmville; Dr.

James Johnston, Staunton ; Dr. Frank A. Jeter, Richmond.

*Committee on Dental Appliances.*—Dr. W. Leigh Burton, Richmond; Dr. J. O. Hodgkin, Warrenton; Dr. R. Y. Henly, King & Queen.

*Executive Committee.*—Dr. Jud. B. Wood, Dr. Frank A. Jeter, Dr. Frank L. Harris.

J. HALL MOORE, *President.*

L. M. COWARDIN, *Cor. Secretary.*

---

### ARTICLE VI.

### *Results of Some Recent Experiments.*

### *Georgia State Dental Society.*

The Tenth Annual Session of the Georgia State Dental Society was held in the City of Atlanta, commencing on the 29th of July, 1878, 10 A. M., President, M. H. Thomas in the chair. The State Beard of Dental Examiners met at the same time and place, and accomplished a very effective work, for the good of the profession throughout the State.

After an unusually interesting session of three days, the Society elected the following officers for the ensuing year, and adjourned to the Second Monday in July, 1879, at Augusta, Ga.

*President.*—Dr. W. C. Wardlaw, Augusta.
1*st Vice President*—Dr. M. S. Johnson, Perry, Ga.
2*nd Vice President.*—Dr. J. A. Chapple, LaGrange, Ga.
*Cor. Secretary.*—Dr. L. D. Carpenter, Atlanta, Ga.
*Rec. Secretary.*—Dr. R. A. Holliday, Atlanta, Ga.

*Treasurer.*—Dr. H. A. Lowrance, Athens, Ga.

*Committee of Arrangements.*—Drs. George Paterson, Waynesboro; G. H. Winkler, Augusta, Ga; Samuel Hape, Atlanta, Ga.

L. D. Carpenter, *Cor. Secretary.*

As the Southern Dental Association commences its Annual Session on the following day, the Second Tuesday in July, the members of the Georgia Society will have an opportunity to attend the meetings of the Association likewise. —Editors Amer. Jour. of Dental Science.

### To Readers and Correspondents.

Communications intended for publication, and exchange journals and papers, should be addressed to the Editor of the American Journal of Dental Science, 259 N. Eutaw St., Baltimore, Md. All letters relating to the business of the journal, or containing cash remittances, to be directed to Messrs. Snowden & Cowman. Articles for publication should be received by the editor at least three weeks previous to the first day of the month on which the number in which it is designed for them to appear, is issued.

### MONTHLY SUMMARY.

☞ The American Journal of Dental Science will contain fifty pages of reading matter in each number, making a volume of about

*Six Hundred* pages

EXCLUSIVE OF ADVERTISEMENTS

THE

### AMERICAN JOURNAL

OF

### DENTAL SCIENCE.

The *Journal* is issued on the first of each month and contains not less than *fifty* pages of reading matter in each number, exclusive of advertisements, making a volume of *six hundred* pages per annum.

In each number will be found Original Communications, Correspondence, Selected Articles, Monthly Summary, Bibliographical Notices and Editorial Matter, and every effort will be exerted to render the *Journal* worthy of the patronage of the Dental profession.

A generous co-operation is respectfully solicited from the members of the profession; and as the *Journal* has now a printing office of its own, which materially lessens the cost of its publication, it will be issued at the reduced subscription price of

**$2.50 Per Annum in Advance.**

Communications are respectfully solicited on all subjects pertaining to the practice of dentistry, as well as reports of cases occurring in dental practice.

### RATES OF ADVERTISING.

| | 1 MO. | 2 MO. | 3 MO. | 6 MO. | 12 MO. |
|---|---|---|---|---|---|
| 1 PAGE. | $15 00 | $22 50 | $30 00 | $50 00 | $100 00 |
| ½ " | 10 00 | 15 00 | 20 00 | 30 00 | 50 00 |
| ¼ " | 7 00 | 11 00 | 15 00 | 20 00 | 30 00 |
| ⅛ " | 5 00 | 8 00 | 11 00 | 15 00 | 20 00 |

All original communications designed for publication, works for review, new instruments for notice, exchanges, &c., should be directed to the editor, 259 N. Eutaw St., Baltimore, Md.

All advertisements and subscriptions should be addressed to

SNOWDEN & COWMAN,

*Publishers of the Am. Journal of Dental Science.*

86 W. FAYETTE ST. BET. CHARLES & LIBERTY STS.,

BALTIMORE, MD

